# Intramedullary implant exchange and adequate soft tissue coverage in exposed implants following fracture related infection in operatively treated ankle fractures

**DOI:** 10.1016/j.tcr.2025.101206

**Published:** 2025-05-26

**Authors:** R.B. Beks, P. van Oosten, D. van Embden, M. Botman, T. Schepers

**Affiliations:** a Amsterdam UMC, Trauma Unit, Meibergdreef 9, PO Box 22660, The Netherlands; b Amsterdam UMC, Department of Plastic Surgery, Meibergdreef 9, PO Box 22660, The Netherlands

**Keywords:** Intramedullary screw fixation, Fracture related infection, Implant exchange

## Abstract

Ankle fractures are common with an increasing incidence due to aging populations. A major complication after surgery for ankle fractures is deep infection, which requires additional treatments and adversely affect long-term outcomes. Treatment of fracture-related infections focuses on achieving fracture union and may involve either retaining or removing the implant, depending on the stability of the implant, with early involvement of a plastic surgeon crucial for adequate tissue coverage. This study presents four cases demonstrating the successful use of intramedullary screw fixation of the fibula to stabilize partially healed fractures, reduce implant load and improve soft tissue coverage in patients with fracture-related infection after operatively treated ankle fractures. Therefore, exchange to intramedullary screw fixation should be considered in patients with fracture related infection of the fibula to reduce the bacterial load of foreign material and allow for better soft tissue coverage and healing.

## Introduction

Fractures of the ankle are the third most common skeletal fracture of the lower limb with an incidence rate of 120–170/100,000 persons per year [[1], [2], [3], [4]]. The most common injury pattern is a simple fall and women are affected more than man [5]. Risk factors for ankle fractures are age-related skeletal fragility and a higher risk of simple falls in the elderly, therefore, due to increasing life expectancy higher incidence rates can be expected over the years [5].

The most important complication after surgery for ankle fractures is a deep infection involving the implanted material resulting in the need for additional treatment and worse long term outcome [6]. The overall number of wound complications after surgery varies widely, with deep infections ranging from 1.3 %–9 % [7,8]. In order to uniformly document fracture related infection (FRI) a consensus definition was established [9,10]. Confirmatory criteria for FRI are presence of a fistula, sinus or wound breakdown; purulent drainage from the wound or presence of pus during surgery; phenotypically indistinguishable pathogens identified by culture from at least two separate deep tissue/implant specimens; and presence of microorganisms in deep tissue taken during an operative intervention. Multiple factors contribute to the risk of FRI including patient specific factors (alcohol consumption, diabetic neuropathy, medication use, compliance), trauma related factors (e.g. open fracture) and surgery related factors (timing and duration of surgery, type of material used) [[11], [12], [13]].

The goal of fracture related infection treatment is fracture union and retaining the implant to support the reduction. Treatment regimens for fracture related infections may differ depending on the time of onset distinguishing between early (<2w postoperatively), delayed (3–10 weeks) and late (>10 weeks) onset [14,15]. Different more virulent pathogens may be present in early infections and late onset infections generally involve the formation of a biofilm on the implanted material [14]. Therefore, two different surgical approaches can be distinguished. The first one consists of debridement, antimicrobial therapy and implant retention if stable (DAIR) and the second comprises debridement, antimicrobial therapy and implant removal (when the fracture is healed) or implant exchange (when the fracture is not yet healed or the implant is unstable). Finally, adequate tissue coverage is of paramount importance and requires early involvement of a plastic surgeon.

This study aims to demonstrate the use of intramedullary screw fixation of the fibula to stabilize partially healed fractures and reduce implant load in patients with fracture-related infections after operatively treated ankle fractures, illustrated through four related cases.

## Case presentation

### Case 1

**Fig. 1:**
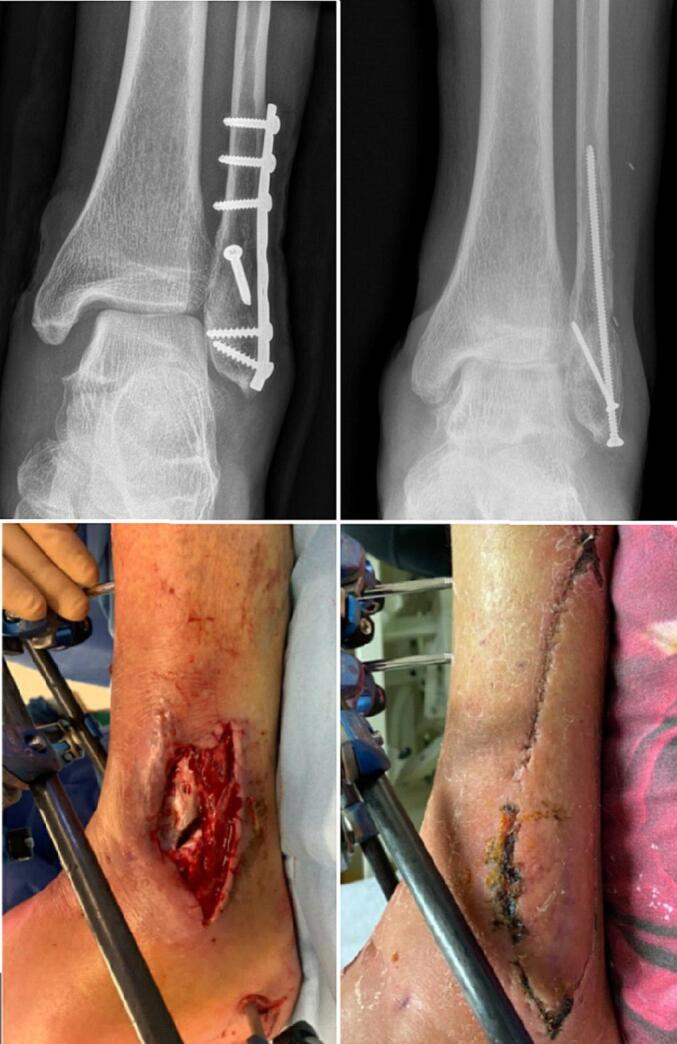
Fracture related infection of the fibula treated with implant exchange and propellor flap.

A 72 year old male suffered a Lauge-Hansen supination-external rotation stage 4 (SE4) Gustillo grade-2 open ankle fracture (Fig. 1). After direct debridement of the medial open wound the fracture was treated with open reduction and internal fixation. After 4 days there was a deep infection which was debrided in the operating room. Two weeks later the implanted material was removed because of ongoing infection and an external fixator was placed. Three weeks after trauma the lateral side of the external fixator was removed and the fibula was addressed with an intramedullary 3.5 mm screw. The remaining lateral soft tissue defect was covered using a propeller flap based on a perforator artery branching of the peroneal artery. The donorsite could be closed primarily. A split skin graft was harvested from the left upper leg and used to cover a superficial skin defect on the medial side of the ankle. The wound cultures showed *Clostridium limosum* and *Staphylococcus haemolyticus*. Antibiotic treatment consisted of intravenous vancomycin and metronidazole for the first week followed by 11 weeks of clindamycin based on cultures. The wounds healed without complications. The medial side of the external fixator remained in place for another 6 weeks.

### Case 2

**Fig. 2:**
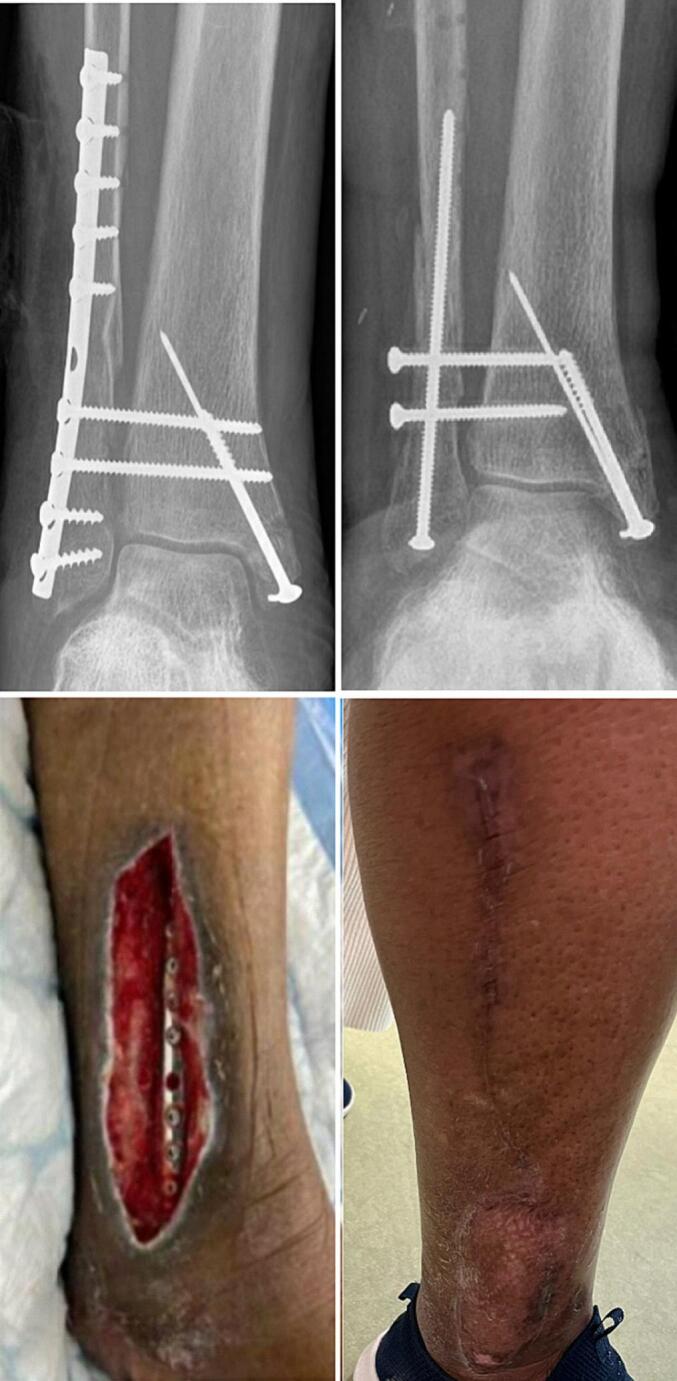
Fracture related infection of the fibula treated with implant exchange and peroneus brevis flap.

A 77 year old woman with a Lauge-Hansen SE4 injury was treated with plate osteosynthesis of the fibula and Kirschner wires for the medial malleolus (Fig. 2). After three weeks she developed a deep infection needing debridement and a negative pressure wound therapy was started. Five weeks later the exposed fibula plate was removed and replaced with a long 3.5 mm intramedullary screw. The remaining skin defect was covered with a vascularized peroneus brevis flap, which was dissected of the fibula from proximal to distal. Different perforator arteries vascularizing the peroneus brevis flap were encountered, of which the most distal perforator was preserved. The peroneus brevis flap was then folded over the defect and a split skin graft was added. The wound cultures showed *Staphylococcus epidermidis*. Antibiotic treatment consisted of intravenous vancomycin and ceftazidime for the first week followed by 11 weeks ciprofloxacin and rifampicin based on cultures. The wounds healed without complications.

### Case 3

**Fig. 3:**
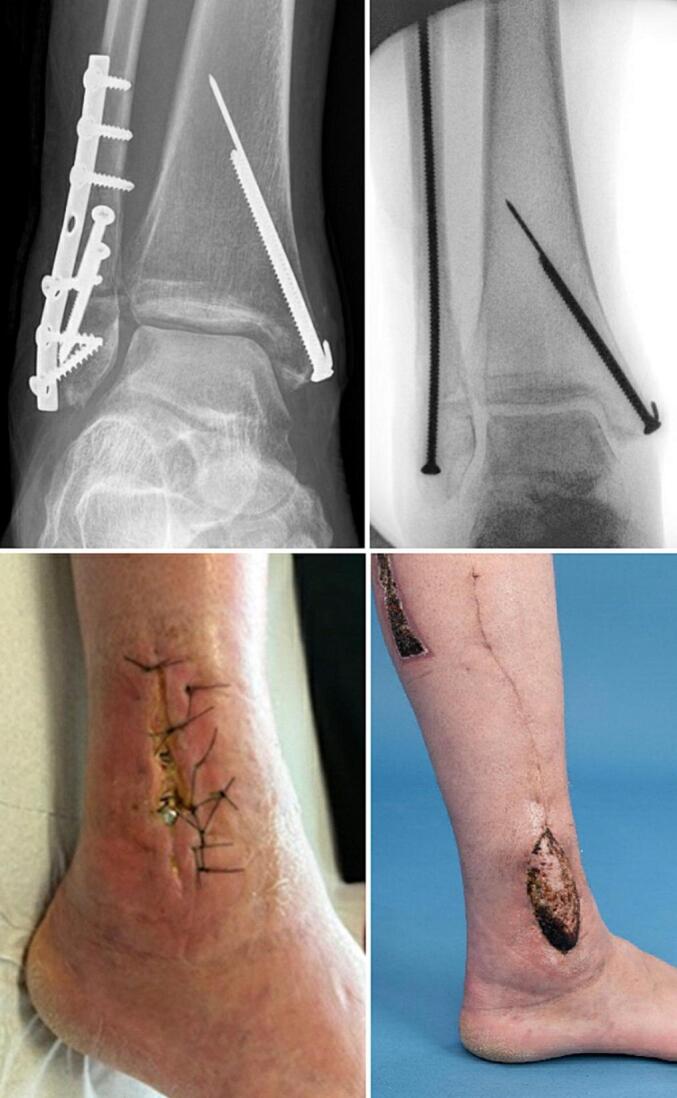
Fracture related infection of the fibula treated with implant exchange and peroneus brevis flap.

A 34 year old woman with a Lauge-Hansen SE4 injury was treated with plate osteosynthesis of the fibula and a combination of screw and Kirschner wire fixation for the medial malleolus (Fig. 3). Two weeks later she developed an FRI and the wound was debrided and closed again assisted with incisional negative pressure wound therapy. Six weeks later the wound was dehiscent with the fibula plate exposed. Thereafter the plate was removed and replaced by a 3.5 mm intramedullary screw in the fibula. The soft tissue defect was covered using a peroneus brevis flap. The flap was dissected from proximal to distal, rotated over the defect and covered with a split skin graft. The wound cultures of the initial debridement showed Acinetobacter calcoacetius-baumannii complex, *Enterobacter cloacae* and *Enterococcus faecalis*. Antibiotic treatment consisted of intravenous vancomycin and gentamycin for the first week followed by 11 weeks clindamycin based on cultures. The wounds healed without complications.

### Case 4

**Fig. 4:**
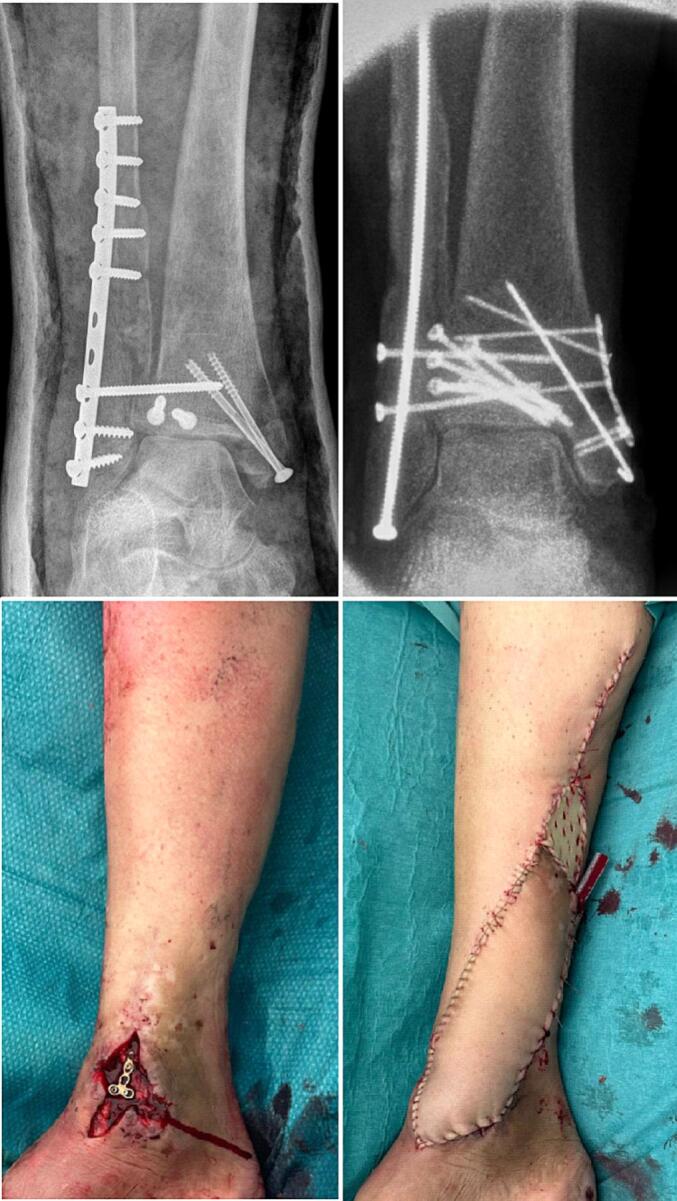
Fracture related infection of the fibula treated with implant exchange and propellor flap.

A 67 year old woman with a Lauge-Hansen SE4 injury was treated abroad with plate osteosynthesis of the fibula and screw fixation for the medial malleolus and malleolus tertius (Fig. 4). Eight weeks postoperatively she presented with medial open wound and multiple fistula in the lateral wound and a malreduction seen on the imaging. All implants were removed of which the lateral plate showed loosening, the malreduction of the fibula, posterior malleolus and medial malleolus were corrected. The initial one third tubular plate of the fibula was replaced with an intramedullary fibula screw. The lateral defect could be closed primarily due to the implant exchange. The medial defect was covered with a fasciocutaneous propellor flap on the posterial tibial artery perforator, with a split skin graft for the donorsite. The wound cultures showed *Enterobacter cloacae* complex. Antibiotic treatment consisted of intravenous vancomycin and meropenem for the first week followed by 11 weeks of ciprofloxacin based on the cultures. The wounds healed without complications.

### Surgical technique

Intramedullary screw fixation was performed as previously described by Loukachov et al. [16]. With the difference that in the aforementioned cases the fibula was exposed, in case of additional reduction or instability the fracture was reduced and held with a small Weber clamp. In short, a 5 mm skin incision was made approximately 1 cm below the tip of the fibula. The drill guide is placed at the tip of the fibula in two planes. After opening of the cortex with a drill a 100 to 150 mm 3.5 mm cortical titanium screw was inserted intramedullary. In female patients with a larger diameter canal a 4.5 mm screw is chosen. After testing the syndesmosis one or two additional syndesmotic screws were placed if deemed necessary.

In the patients undergoing soft tissue coverage with a propeller flap, a hand-held Doppler probe was used to locate the perforator artery vascularizing the flap pre-operatively. The flap was then designed around the vascular pedicle of choice, based on the distance of the perforator to the defect and the size of the defect. The perforator was freed from fascia and other tissue over several centimeters all the way to the peroneal artery or the posterior tibial artery, to prevent torsion of the vessels. It was then rotated 180 degrees into the defect and sutured in place without tension. The donor defect was primarily closed.

When the perforators where not ideally positioned in specific patients we opted for a peroneus brevis flap. For dissection of the peroneus brevis flap, the incision around the existing soft tissue defect was extended proximally. The superficial peroneal nerve was identified and spared. Then, the fascia of the lateral compartment was opened, identifying the peroneus brevis muscle and its perforators. The muscle was elevated from proximal to distal over the fibula, ligating several proximal perforators and preserving one or more distal perforator arteries. When sufficient elevation was achieved, the flap was rotated over the defect and covered with a split-thickness skin graft.

## Discussion

This study presents four cases demonstrating the use of intramedullary screw fixation of the fibula to stabilize fractures and reduce implant load in patients with FRI following operatively treated ankle fractures.

Many studies have demonstrated intramedullary screw fixation for the fibula to be adequate and resulting in good outcome [16]. Reduction and biomechanical stability have been shown to be similar in intramedullary screw fixation versus plate and screw fixation of the fibula [17]. Even though open reduction and internal fixation using plate and screw remains the Gold Standard for distal fibula fractures, intramedullary screw fixation has been described to be a suitable alternative in patients with fragile soft tissue or comorbidities to prevent wound healing complications. Additional benefits of the use of intramedullary screw fixation includes the prevention of screw penetration into the ankle joint, as well as avoiding damage to the superficial peroneal nerve and injury to the peroneal tendons [16].

Fracture related infection of operatively treated fibula fractures have been associated with a worse functional outcome [12,18]. Several factors have been identified contributing to the risk of FRI including the timing of surgery [12]. In order to reduce the risk of complications adequate and timely fixation is crucial. Still, with the increasing incidence of ankle fractures, FRI will be commonly encountered in daily practice requiring adequate work-up and treatment. The general work-up for FRI has been described thoroughly but the limited soft tissue surrounding the distal fibula requires extra care with respect to implant exchange and soft tissue coverage. In this case report we demonstrated the successful use of intramedullary screw fixation in patients with FRI after plate fixation resulting in an adequate implant exchange while reducing implant load to control bacterial load and allow for easier soft tissue coverage and healing.

Soft tissue coverage of exposed bone or osteosynthesis material is crucial for preventing and adequately treating FRI's [19]. Minimal tension wound closure is important to avoid wound dehiscence, scarring and necrosis [20]. To cover the defect, it is recommended to choose the simplest reconstructive approach deemed effective, according to the “reconstructive ladder” [21]. Soft tissue reconstruction of the distal third of the leg is particularly difficult due to lack of mobility of the surrounding soft tissues and poor healing [22,23]. For this reason, even small defects, with exposed bone, tendons or osteosynthesis material, often require flap coverage [22].

In small defects around the ankle joint, local coverage options may include fasciocutaneous transposition flaps such as a visor flap (a bipedicled advancement flap), a classic Ponten transposition flap or a VY flap for smaller defects [24]. These flaps, however, are limited in size in this area and have a higher risk of venous congestion [23].Two specific local flaps that became popular in our center over the past years are the propellor flaps and the peroneus brevis flap presented in this article. When these local coverage options do not suffice, free flaps are used. For relatively small defects, like the patients presented in this article, we rarely need a free flap. Free flaps are more invasive but have proven to result in less flap failure, less infections and faster bone healing compared to local coverage, especially for larger defects [22,25]. Size and shape can easily be planned and recipient vessels can be chosen out of the zone of injury (e.g. gracilis, latissimus dorsi and anterolateral thigh) [24].

### Conclusion

Implants exposed after FRI have the potential to sustain infection and therefore may require replacement if no fracture healing has been achieved. Intramedullary screw fixation of the fibula is a fixation method which has been shown to be adequate in maintaining stability of the fibular reduction. Adequate soft tissue coverage may be achieved using a peroneus brevis muscle flap or the fasciocutaneous perforator based propeller flap. In patients with an FRI after plate fixation of the fibula, exchange to intramedullary fixation should be considered to reduce the bacterial load of foreign material and allow for better soft tissue coverage and healing.

## CRediT authorship contribution statement

**R.B. Beks:** Writing – review & editing, Writing – original draft, Conceptualization. **P. van Oosten:** Writing – review & editing, Writing – original draft. **D. van Embden:** Writing – review & editing. **M. Botman:** Writing – review & editing. **T. Schepers:** Writing – review & editing, Writing – original draft, Conceptualization.

## Declaration of competing interest

The authors declare that they have no known competing financial interests or personal relationships that could have appeared to influence the work reported in this paper.
